# Streamlining the study start up process

**DOI:** 10.1186/1745-6215-14-S1-P140

**Published:** 2013-11-29

**Authors:** Samantha Wileman, John Norrie

**Affiliations:** 1 University of Aberdeen, Aberdeen, UK

## 

Trial start up is a frenetic time for any trial manager: once a trial has been funded the clock is ticking to get the first participant recruited. There are a number of key activities that need to happen during this stage, so developing standardised processes to help improve effectiveness can make the system more efficient, and help promote best practice. Three of the key processes that have been developed within our CTU have been:

• a centralised resource repository, housing a suite of trial paperwork templates (e.g. consent forms, trial master file checklists, case report forms etc), which can be easily customised to each new trial. This has proven to be of great value to new trials.

• the use of specialised quality management software to manage and control these templates, thus ensuring version control and a managed (and auditable) approach to template upgrading. In addition to any revisions made following legislative or regulatory changes, all staff are encouraged to raise ‘change requests' based on their experiences of using these templates to ensure continued improvement.

• a bespoke electronic trial filing structure. Most of the trials managers work across more than one trial so a secure and systematic approach to managing the day-to-day trial documentation has not only ensured consistency in trial conduct but has been highly advantageous during inspections/monitoring visits.

This talk will use examples from real trials to illustrate these processes.

